# A Comparison of the Low-FODMAPs Diet and a Tritordeum-Based Diet on the Gastrointestinal Symptom Profile of Patients Suffering from Irritable Bowel Syndrome-Diarrhea Variant (IBS-D): A Randomized Controlled Trial

**DOI:** 10.3390/nu14081544

**Published:** 2022-04-08

**Authors:** Francesco Russo, Giuseppe Riezzo, Antonella Orlando, Michele Linsalata, Benedetta D’Attoma, Laura Prospero, Antonia Ignazzi, Gianluigi Giannelli

**Affiliations:** 1 Functional Gastrointestinal Disorders Research Unit, National Institute of Gastroenterology “S. de Bellis” Research Hospital, 70013 Castellana Grotte, BA, Italy; giuseppe.riezzo@irccsdebellis.it (G.R.); antonella.orlando@irccsdebellis.it (A.O.); michele.linsalata@irccsdebellis.it (M.L.); benedetta.dattoma@irccsdebellis.it (B.D.); laura.prospero@irccsdebellis.it (L.P.); antonia.ignazzi@irccsdebellis.it (A.I.); 2 Scientific Direction, National Institute of Gastroenterology “S. de Bellis” Research Hospital, 70013 Castellana Grotte, BA, Italy; gianluigi.giannelli@irccsdebellis.it

**Keywords:** irritable bowel syndrome, low-FODMAPs diet, symptom profile, tritordeum

## Abstract

The dietary approach low in oligosaccharides, disaccharides, monosaccharides, and fermentable polyols (FODMAPs-LFD) is a good strategy for treating irritable bowel syndrome (IBS). Beyond the LFD, other dietary approaches with beneficial effects may be hypothesized. Among them, consumption of Tritordeum-based foods (TBD, bread, bakery products, and pasta) in substitution of other cereals seem to achieve promising results. In a randomized controlled trial, we compared the effects of 12 weeks of LFD to TBD in improving the symptom profile of IBS-diarrhea (IBS-D) patients. The two diets equally improved gastrointestinal symptoms and QoL, measured by the IBS Severity Scoring System (IBS-SSS) questionnaire, reducing the total score after four weeks and maintaining this range until the end of treatment (IBS-SSS total score change: −132.1; 95% CI: −74.9 to −189.4 and −130.5; 95% CI: −73.2 to −187.7; *p* < 0.0001 after LFD and TBD, respectively). The two diets did not modify the micronutrients content when extended for 12 weeks. LFD could be regarded as a first-line dietary approach for IBS-D patients. However, TBD may represent a valid alternative, with high palatability, especially among Italian patients, for whom pasta is considered one of the main assets of dietetic culture, and would be easier to manage in their daily habits.

## 1. Introduction

Irritable bowel syndrome (IBS) is a functional, non-organic gastrointestinal (GI) disorder that significantly affects the quality of life (QoL) of about 5–10% of the adult population in Western countries [1,2]. Without reliable biochemical or instrumental markers, IBS is defined by the presence of distinctive symptoms (discomfort or recurrent abdominal pain with modifications in the stool habit), described by the Rome criteria [3]. IBS patients can be categorized into subgroups based on the predominant bowel pattern (IBS-C: defecation with constipation; IBS-D: defecation with diarrhea; IBS-M: a mixture of constipation and diarrhea) [4]. The clinical management of this syndrome is still mainly symptomatic, with the therapeutic goal primarily being to reduce symptoms and improve the patients’ QoL.

The severity of symptoms complained of by patients significantly affects the choice of adopted treatment. In less severe cases, the use of effective anti-stress therapy, the constant practice of physical exercise, an appropriate night’s rest, and a dietary plan excluding foods causing typical IBS symptoms could, at least, mitigate this condition. In moderate–severe cases, adding “ad hoc” drug therapy and, sometimes, psychotherapeutic support may become necessary [5]. Additionally, probiotics and prebiotics supplements can only be administered for a limited period due to loss of long-term adherence and their high cost.

A structured dietary approach could represent a reliable alternative strategy. It is now well-established that many people with IBS-D often describe worsening symptoms after eating certain foods, such as those containing fermentable oligosaccharides, disaccharides, monosaccharides, and polyols (FODMAPs). These short-chain carbohydrates are present in some foods, including wheat and beans, and may contribute to the onset of different digestive symptoms such as bloating, abdominal pain, and diarrhea due to poor absorption in the small intestine. In this way, FODMAPs are digested by colonic bacteria, releasing gas in the colon. Furthermore, FODMAPs can act as osmotic agents, increasing the water volume in the stool [6]. In recent years, our group has focused research on the properties of a low-FODMAPs diet (LFD) in the clinical management of patients with IBS-D, demonstrating that these patients benefit from a 12-weeks LFD, which can mitigate the symptoms, reduce the inflammatory status, increase the vitamin D content, and affect the lipidomic profile [7,8]. Unfortunately, adherence to an LFD can be somewhat problematic, needing continuous nutritional support. Beyond the LFD, other dietary approaches with beneficial effects may be hypothesized. Among them, alternative grains such as Tritordeum seem to achieve good results. Tritordeum is a cereal derived from the hybridization of durum wheat and wild barley. Originally cultivated with traditional techniques in Spain, it has more recently grown in Apulia, a region in southern Italy. Tritordeum grows well with little care, even in adverse conditions. Notably, this cereal has significantly lower levels of gliadins, fewer carbohydrates and fructans, and a higher content of dietary fibers, proteins, and antioxidants than classic wheat [9]. The presence of gluten makes this cereal unsuitable for celiac disease (CD) patients, but it could be appropriate for preparing foods for those patients with non-celiac wheat sensitivity (NCWS) or IBS-D [10] that mainly complain of abdominal bloating and could benefit from removing wheat from the diet due to its gluten and fructans content.

The multifaceted setting of nutrients in wheat-based foods, other than gluten [11,12], could account for the different IBS-D symptoms. It has already been postulated that some monococcal diploid grain lines, with minimal activation of the innate immunity and a reduced amount of toxic gluten peptides, could be helpful for IBS-D patients.

For these reasons, our group recently performed a pilot study to investigate the effects of a 12-week diet with Tritordeum-based foods (TBD- bread, bakery products, and pasta) in substitution of other cereals on the GI symptoms and the status of the GI barrier in IBS-D patients. TBD proved to significantly reduce IBS-D patients’ symptoms by affecting intestinal permeability [13]. Thus, the purpose of this study was to evaluate, in a randomized controlled trial, the beneficial effects of 12 weeks of LFD compared with TBD for the same duration on improving the symptom profile of IBS-D patients.

## 2. Materials and Methods

The protocol of the study has already been published [8]. The methodology is summarized below.

### 2.1. Study Design and Participants Enrollment

A multi-disciplinary, single-blind, parallel-group randomized controlled trial design was conducted from January 2018 to September 2020 with patients diagnosed with IBS-D according to Rome III-IV criteria [3] in the Functional Gastrointestinal Disorders Research Unit-National Institute of Gastroenterology “S. de Bellis” Research Hospital (Castellana Grotte, Italy). The present study is part of the broader research on alternative nutritional strategies for IBS-D patients based on diets with a low content of short-chain carbohydrates compared to specific IBS dietary advice. The trial protocol was approved by the Scientific Committee of IRCCS “S. de Bellis” and the Ethics Committee of IRCCS “Ospedale Oncologico–Istituto Tumori Giovanni Paolo II”, Bari, Italy, (N. 274/C.E. 12.12.17). All procedures were conducted in compliance with the World Medical Association Declaration of Helsinki, the Guidelines of the International Conference on Harmonization on Good Clinical Practice, and the Ethical Conduct for Research Involving Humans [14]. We adhered to the Consolidated Standards of Reporting Trials (CONSORT) guidelines for reporting on randomized clinical trials [15,16]. Before participants were enrolled in the study, consent to collect clinical, nutritional, and analytical data was obtained in writing.

Recruitment was carried out by online bulletin posts, flyers available in physician offices, and the placement of posters at our Institute.

The exclusion criteria were GI symptoms with a total score lower than 125 on IBS-Severity Scoring System (IBS-SSS) [17], celiac disease, wheat allergy, organic GI disease, severe organic and psychiatric diseases, or already on a diet (i.e., LFD, gluten-free diet, vegan diet). The lactose-reduced diet was allowed for subjects with lactose intolerance, but patients had to agree to keep this intake constant during all diet periods. Antipsychotic drugs and medications interfering with GI motility, weight, and appetite were excluded. Patients also had to change their current diet to enter the study.

Patients were randomly allocated to receive either the LFD or TBD for 12 weeks after exclusion criteria were applied. Randomization was carried out using a computer-generated allocation schedule performed by an investigator not actively participating in the study. All the patients were blinded to the type of intervention.

**Figure 1:**
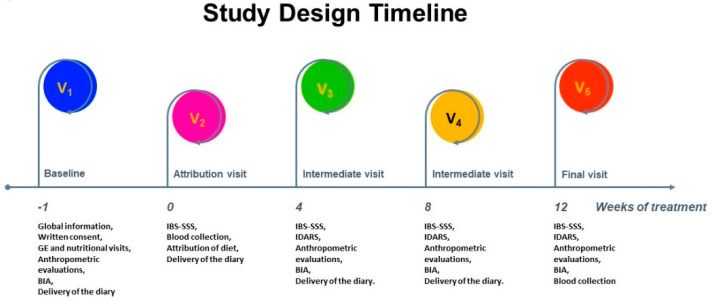
The study design timeline. GE = gastroenterological, BIA = bioelectrical impedance analysis, IBS-SSS = Irritable Bowel Syndrome Severity Scoring System, IDARS = IBS diet adherence reporting scale.

The study design timeline was structured over five visits and has already been described elsewhere [7] (Figure 1).

Briefly, at baseline (V_1_), subjects underwent a physical examination by a gastroenterologist and an interview with a trained nutritionist. Anthropometric measurements and bioelectrical impedance analysis (BIA) were performed. Eligible patients ate a run-in diet for two weeks and recorded their food intake. The bowel habits were reported according to the Bristol stool table chart. In addition, their physical activity and medication use were recorded to accurately determine daily intake and energy expenditure. One week after V_1_, all patients underwent a second visit (V_2_), during which they completed the IBS-SSS questionnaire [17]. They had to receive an IBS-SSS total score >125 to be recruited. Eligible subjects were then placed on one of two diets (LFD or TBD), and they were also asked to fill out a daily diary until the end of the diet. They also provided stool, urine, and blood samples.

During the follow-up period (V_3_ at week four and V_4_ at week eight), symptom and diet diaries completed in the previous weeks were collected, and patients compiled the new IBS-SSS and the IBS Diet Adherence Reporting Scale (IDARS). This questionnaire consists of five questions about dietary compliance, with scores ranging from 1 to 5 for each item. A total score of at least 20 indicates good adherence to the diet [18]. BIA and anthropometric measurements were also performed. After 12 weeks on the prescribed diet (V_5_), the researchers collected the symptom and dietary diaries compiled in the previous weeks. Patients completed IBS-SSS and nutritional diaries to assess their adherence to the recommended diet (IDARS) and underwent the same clinical, anthropometric, and analytical measurement procedures as V_2_.

### 2.2. GI Symptom Scores

Symptom characteristics were assessed using the validated GI symptom questionnaire IBS-SSS [16]. The questionnaire provides an overall measure of symptom severity by assessing five items (“Severity of abdominal pain”, “Frequency of abdominal pain”, “Severity of abdominal distension”, “Dissatisfaction with bowel habits”, “Impairment of QoL”) on a visual analog scale. Each symptom is scored on a 100-point scale. For four items, patients marked a point on the line representing how they felt, and the proportional distance from zero (ranging from 0 to 100) represented the item’s score. The fifth item asked the number of days out of ten when subjects complained of abdominal pain. The answer was multiplied by 10 to create a metric scale from 0 to 100. The five items were added together for a total score between 0 and 500. Cases were classified as mild (75 to 175), moderate (175 to 300), and severe (>300). Healthy subjects conventionally have a score below 75, whereas patients with scores lower than 75 are considered in remission.

### 2.3. LowFODMAPs Characteristics and Diet

A personalized LFD was assigned after reviewing a food diary and having a one-on-one personal consultation with a nutritionist during V_2_. The rationale for LFD stands in limiting the intake of foods containing fermentable oligosaccharides, monosaccharides, disaccharides, and polyols [6]. Daily macronutrient intake (50% glucose, 30% lipid, and 20% protein) was calculated using dedicated software (Nutrigeo Software 8.6.0.0, Progeo Medical, Centobuchi di Monteprandone, AP, Italy). Diet is an appropriate and individualized dietary plan matching basal metabolic rate and daily energy expenditure results from population studies with anthropometric data. Participants were given a detailed weekly structured menu consisting of three meals (breakfast, lunch, and dinner) and two snacks (morning and afternoon). Patients also received a booklet detailing what foods are allowed, which foods to avoid and which foods to reduce, based on the classifications used by Monash University and the cut-off values for each FODMAPs subgroup [18,19,20]. Nutritionists created a leaflet for patients in the study, with details on where to buy specific products. Additionally, nutritionists guaranteed adequate fiber intake, and also offered advice on cooking without onions and garlic and other high-FODMAPs foods. Drinking alcohol was not recommended, although it is not high in FODMAPs.

### 2.4. Tritordeum-Based Foods Characteristics and Diet

As for TBD, all the food items (bread, pasta, “taralli” (local salty biscuits), and breakfast biscuits) were prepared using Tritordeum flour. The energy content and chemical composition of Tritordeum flour and pasta are represented in Table 1.

Table 2 reports the ingredients and preparation of Tritordeum-based foods used in the study. A controlled TBD was provided to each patient. The daily menu was breakfast, mid-morning snacks, lunch, afternoon snacks, and dinner. This intervention diet implied that each patient in the study had to consume flour, bread, breakfast biscuits, taralli, and pasta prepared exclusively with Tritordeum. The diets were prepared as described elsewhere [13], by matching basal metabolism and daily energy consumption with anthropometric data for all patients to assign suitable and tailored dietary regimens. The software utilized to assess the daily intake of macronutrients (50% carbohydrates, 30% lipids, and 20% proteins) was the same as described for LFD.

### 2.5. Assessment of Nutrient Intakes

Throughout the study, patients followed a controlled diet and abstained from alcohol or engaging in vigorous physical activity.

Trained staff provided the dietary instructions and interviewed each subject before starting the diet to obtain a report on their usual diet and calculate energy requirements (see V_1_ in Study Design). Patients kept food diaries to assess their energy intake and expenditure during the study period. Energy intake refers to the calorie intake with food and drinks per unit time (24 h). Energy expenditure is the total energy expenditure consumed by the organism within a unit of time (24 h) to maintain its structural and functional properties and perform physical activities. Along with the description of physical activity and its duration, the diary contained the quantities (in grams) and descriptions of foods eaten each day for breakfast, lunch, dinner, and snacks.

Nutritionists then examined food diaries that were completed at baseline and throughout the diet. Special software was used (Progetto Dieta v. 2.0—www.progettodieta.it—last accessed 18 January 2022). In addition, the daily energy intake and the intake (kcal) of carbohydrates, lipids and protein (in percent and weight), alcohol consumption (in percent), and dietary fibers (in grams) were calculated.

### 2.6. Biochemical Analyses

Blood samples were withdrawn between 8:00 and 9:00 a.m., following a 10–12 h overnight fast. All samples were analyzed by the laboratories immediately or during the first week of the collection after freezing at −80 °C. Metabolic parameters were glucose, total cholesterol (TC), low-density lipoprotein cholesterol, high-density lipoprotein cholesterol (HDL-C), the TC/HDL-C ratio, and triglyceride. Other serum biochemical parameters were: C-reactive protein (CRP), urea, creatinine, iron, urate, cobalamin and folate, calcium and phosphorus, parathyroid hormone, vitamin D, and total serum protein. All parameters were assessed by commercial kits.

### 2.7. Statistical Analysis

A per-protocol analysis was performed using only participants who completed all testing timepoints (i.e., patients who dropped out after intermediate evaluations were excluded from the secondary analysis) using SPSS for Windows (version 22.0; SPSS Inc., Chicago, IL, USA). Continuous variables were presented as mean ± SD, except in figures, where mean ± SEM was used for clarity. For between-group comparisons at baseline, Student’s *t*-test was used. A mixed-design ANOVA was performed to examine the differences in the outcome variables between groups and before and after treatment. Mixed-design ANOVA consists of a mix of one between-subjects factor (LFD and TBD groups) and one within-group factor (before and after treatment-V_2_ to V_5_) to see whether the treatment led to any change in a given variable. Specifically, a significant main diet effect meant significant differences in the clinical and biochemical variables between the two diets. A significant main “Time” effect meant the presence of significant differences between the repeated measures, irrespective of diet membership (V_2_ vs. V_5_). Lastly, a significant “interaction effect” (Diet x Time) meant significant differences between the groups and over time, e.g., the change in symptom scores and biochemical variables over time was different depending on group/diet membership. In addition, for each ANOVA result that was significantly different after the intervention diet, pairwise comparisons were performed using a post hoc Bonferroni analysis to identify where the differences occurred.

For the sample size calculation, a probabilistic error of type I (α = 0.05) and type II (β = 0.80), a standard deviation of the IBS-SSS score equal to 80, and a correlation of 40% between the different measures were considered. The effectiveness of the two diets (LFD and TBD) in controlling symptoms was also taken into account, as assessed by the reduction in the IBS-SSS total score after 12 weeks of treatment. These data were derived from our previous published [8] and unpublished observations. Based on these findings, the expected difference in the efficacy in improving symptoms between the two diets was set at 75 score points. This score provided a sample size of 19 patients for each group. Based on a putative loss for each experimental arm of about 45%, a sample size of at least 28 patients per arm was considered for recruitment.

## 3. Results

### 3.1. Number and Characteristics of the Patients

**Figure 2:**
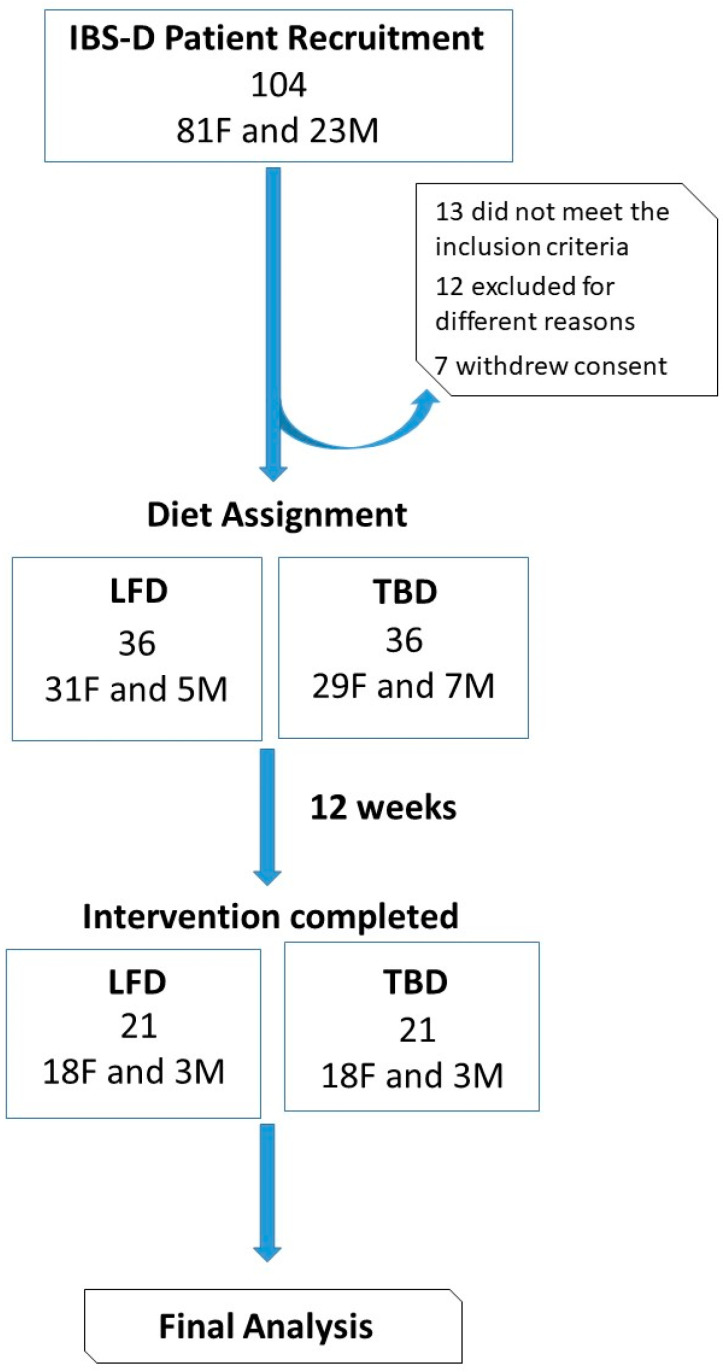
The CONSORT flowchart of the study.

Figure 2 summarizes the flow of the patients through the study. One hundred and four patients with IBS-D, 81 females (F), and 23 males (M), entered the study. Of these patients, 13 did not meet the inclusion criteria, 12 were excluded for different reasons (pregnancy, abdominal surgery, use of antibiotics, moved to other districts, change in working activities), and 7 withdrew consent. Seventy-two IBS-D (58 F and 14 M) patients were randomized to the LFD or TBD for 12 weeks. The percentages of patients who did not complete the intervention in the two groups were similar. Twenty-one (18 F and 3 M) patients in the LFD group and 21 (18 F and 3 M) in the TBD group completed the intervention.

Following the CONSORT guidelines, baseline data for randomized individuals (namely, anthropometric values, BIA measurements, and biochemical characteristics) were comparable in both diet groups.

### 3.2. The Symptom Profile in IBS-D Patients

**Figure 3:**
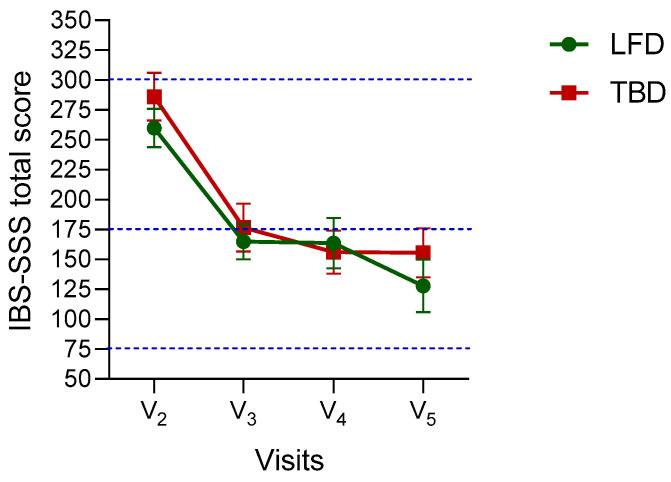
The effects of diets during administration on the IBS Severity Scoring System (IBS-SSS) total scores in IBS-D patients. Mixed-design ANOVA analysis and Bonferroni’s multiple comparisons test. V_2_ (day 0): attribution visit; V_3_ (4 weeks) and V_4_ (8 weeks): intermediate visits; V_5_ (12 weeks): final visit. LFD: Low-FODMAPs diet. TFD: Tritordeum-based food diet. Dotted blue lines indicate scores categorizing IBS symptoms as “mild” (75 to 175), “moderate” (175 to 300), and “severe” (>300). Data are expressed as M±SEM. Multiple comparisons IBS-SSS total score change: −132.1 (95% CI: −74.9 to −189.4) and −130.5 (95% CI: −73.2 to −187.7) after LFD and TBD, respectively (*p* < 0.0001).

Figure 3 shows the effects of diets according to the IBS-SSS total score in IBS-D patients, considering all the timepoints in the study design (from V_2_ to V_5_). IBS-SSS total score deeply decreased after four weeks of treatment, with a further but less dramatic reduction in the following weeks. No difference was found between groups. In more detail, in the case of LFD changes in total recorded IBS-SSS score V_3_ (4 weeks) (−95.0; 95% CI:−37.7 to −152.3), V_4_ (8 weeks) (−96.38; 95% CI: −39.1 to −153.6), and V_5_ (12 weeks) (−132.1; 95% CI: −74.9 to −189.4) were significantly different regarding the total recorded IBS-SSS score at V_2_ (baseline) (for all the comparisons, *p* < 0.0001). After TBD, changes in IBS-SSS total score recorded at V_3_ (4 weeks) (−109.5; 95% CI: −52.2 to −166.7), V_4_ (8 weeks) (−129.2; 95% CI: −72.0 to −186.5), and V_5_ (12 weeks) (−130.5; 95% CI: −73.2 to −187.7) were significantly different regarding the total recorded IBS-SSS score at V_2_ (baseline) (for all the comparisons, *p* < 0.0001).

Table 3 reports the IBS-SSS single item. The ANOVA highlighted a time effect without difference between groups. Specifically, “Severity of abdominal pain” reduced by −25.8 (95% CI −10.3 to −41.3; *p* < 0.0001) after 12 weeks of LFD, and −24.2 (95% CI: −8.7 to −39.8; *p* < 0.0001) after 12 weeks of TBD; “Frequency of abdominal pain” reduced by −26.7 (95% CI: −9.8 to −43.6; *p* < 0.0001) after LFD and −23.7 (95% CI: −6.8 to 40.6; *p* = 0.0007) after TBD; “Severity of abdominal distension” reduced by −28.1 (95% CI: −10.2 to −46.0; *p* < 0.0001) after LFD and −30.9 (95% CI: −13.0 to −48.7; *p* < 0.0001) after TBD; “Dissatisfaction with bowel habit” reduced by −27.9 (95% CI: −8.0 to −47.7; *p* = 0.0006) after LFD and −28.2 (95% CI: −8.3 to −48.0; *p* = 0.0005) after TBD; “Interference with quality of life” reduced by −23.7 (95% CI: −6.1 to 41.3; *p* = 0.0012) after LFD, and −23.4 (95% CI: −5.8 to −41.0; *p* = 0.0015) after TBD.

Table 4 reports the anthropometric and the bioelectrical impedance (BIA) measurements before (V_2_) and after 12 weeks of the two diets (V_5_), respectively.

After the intervention, a time effect was found without a difference between the two diets concerning the anthropometric and BIA parameters. Different anthropometric items significantly decreased, such as weight, body mass index (BMI), abdominal and waist circumferences, along with BIA parameters such as fat mass (FM), free fat mass (FFM), total body water (TBW), and Extracellular water (ECW) (Table 4).

Table 5 reports the biochemical measurements before (V_2_) and after 12 weeks of the two diets (V_5_). The ANOVA highlighted that no effects on lipid profile were found at the end of treatments. Following the intervention, fasting glucose and CRP values significantly (*p* < 0.05) decreased over time, with no significant difference between the two diets. By contrast, vitamin D concentrations (*p* = 0.04) increased over time, with no difference between groups per administration time. Lastly, no differences were found in circulating concentrations of iron, serum protein, vitamin B12, folate, and creatinine, indicating that both diets did not affect the micronutrient profile (Table 5).

## 4. Discussion

The present research aimed to evaluate, in a randomized controlled trial, the effects of 12 weeks of TBD compared with LFD of the same duration in improving the symptom profile of IBS-D patients.

The two diets equally improved GI symptoms and quality of life as measured by the IBS-SSS questionnaire, reducing the total score significantly after four weeks and maintaining this range until the end of treatment. In addition, the two diets did not modify the micronutrient content. The present data confirm the validity of LFD for treating IBS-D patients. However, TBD may represent a valid alternative, especially among Italian patients, for whom pasta is considered one of the main assets of dietetic culture, and could be easier to manage in their daily habits.

The pathophysiology of IBS is quite complex, involving a combination of motility disorders, visceral hypersensitivity, altered mucosal and immune function, altered gut microbiota, and disturbed central nervous system processing. Moreover, food plays a considerable role [21,22]. Many popular, healthy foods have a high-FODMAPs content, including dairy products, grains (wheat and rye), fruits (apples, pears, or peaches), vegetables (mushrooms, onions, and garlic), and sweeteners (sorbitol) [23].

FODMAPs are osmotically active molecules that reach the colon unabsorbed, inducing colonic bacteria to ferment them rapidly [24,25,26,27,28]. These events lead to distension and faster oro-cecal transit time, causing bloating, flatulence and diarrhea [29]. Due to visceral hypersensitivity, the same magnitude of distension will produce different intensities of symptoms, depending on individual susceptibility [30].

There is now broad consensus on the efficacy of LFD in improving symptoms in IBS-D patients. Studies prolonged over 12 months demonstrated that LFD should be continued for at least three months to exert the most efficacy. The best improvement in symptoms was seen in patients who were fully adherent to LFD [31]. However, LFD could be tiresome to follow, due to the constant need for the support of a trained dietician and a careful restriction of otherwise healthy foods. Thus, a more straightforward approach, substituting only wheat-based foods with food based on a less immunogenic cereal such as Tritordeum, could represent an alternative strategy.

In recent years, increasing attention has been paid to the role of wheat and other cereals in IBS-D. Some IBS-D patients may experience GI and extra-GI symptoms, exacerbated by the ingestion of cereals. While gluten is most responsible for CD pathogenesis, along with the involvement of innate and adaptive immunity in the response against the prolamins of cereals [11], wheat-based foods can show a variety of nutrients other than gluten, that could be responsible for the IBS-like symptoms with the involvement of innate immunity alone, at least for NCWS [8].

It has already been hypothesized that some monococcal diploid grain lines, characterized by a minimal activation of the innate immunity and a reduced amount of toxic gluten peptides, could also be relevant for other GI conditions, mainly IBS. Ours and others’ results still demonstrated that patients with IBS-D and IBS-M who generally suffer from abdominal bloating as the dominant symptom seemed to improve their symptom profile by eliminating wheat from their diet [9] or using Tritordeum-based food [13].

Upon entry into the study, the two groups of patients were comparable in symptoms. Both groups showed an IBS-SSS total score identifying patients suffering from moderate symptoms. Similarly, nutritional and biochemical variables were comparable between the two groups.

The dietary treatments were administered for twelve weeks and compared for symptom improvement. We used a uniform, consensus-based, comparable measurement of symptom severity (e.g., IBS-SSS), consisting of five questions that measure “Severity of abdominal pain”, “Frequency of abdominal pain”, “Severity of abdominal distension”, “Dissatisfaction with bowel habits”, “Interference with QoL” on a 100-mm visual analog scale. This score measures overall IBS severity and QoL [17]. Beyond previously published data from ours and other groups on the need for at least 12 weeks of treatment, this duration was adopted since it could reflect better the effects on GI symptoms than the usual four-week diet, potentially recording all the natural fluctuations in the symptom profiles occurring in IBS-D patients, irrespective of diet.

The patients reported full compliance to both diets for the study duration. The pre- and post-intervention scores between the LFD and TBD were assessed.

The most significant finding from our study was that both diets were effective in improving the GI symptoms of IBS-D patients, as demonstrated by the absence of significant differences in the total IBS-SSS score at the end of treatment between the two groups. Following the intervention, the LFD group showed a similar decrease in the total score to that of the TBD group.

The observed improvement was at least 70-75 points, undoubtedly a better value than that expected by other authors, who set 50 points as a desirable primary outcome [32].

The drastic decrease from “moderate” to “mild” in the total score occurred after four weeks of administration (from V_2_ to V_3_) for both diets, even showing a further slight decrease at V5 compared to the baseline for LFD. This result agrees with all the observations of improvement following short-term treatment. However, independently from the dietary approach, the duration of diets appeared to be statistically related to a patient’s improvement in GI symptoms. This evidence seems to agree with the mentioned importance of the treatment duration. A longitudinal assessment of the study showed that patients from both groups still experienced improvements in the GI symptom profile at the V_5_, 12 weeks after the start of the intervention, like that recorded at intermediate visits.

Furthermore, all the different items that make up the IBS-SSS showed an evident improvement over time (namely, the intensity and frequency of abdominal pain, the intensity of abdominal bloating, dissatisfaction with the intestinal habit, and the interference with QoL) following a similar trend to that of the IBS-SSS total score. This result appears to be directly linked to diet rather than representing a consequence of the placebo effect that, indeed, would not have persisted for such a long time.

Outside the setting of a research intervention, LFD is likely to be followed by those individuals with an increased account of the health benefits of appropriate dietary patterns. However, the avoidance of such different food groups may be unsustainable over a more extended period. In this regard, TBD could represent a more bearable effort to be sustained, especially considering the typical Italian diet, based on pasta consumption, and the higher compliance offered for our patients from this type of diet. Firstly, the palatability is comparable to that of normal pasta, with undoubted advantages for compliance with the diet in the long term. Secondly, the economic burden that the patient has to bear to follow the LFD compared to the TBD could limit adherence to that diet. One strategy could be based on using LFD in the early stages to verify a positive response from those patients for whom an intolerance to these substances is assumed. TBD could instead be hypothesized in the long-term management, especially in our territorial context.

It might be argued that the evaluation of symptoms could be strongly affected by a placebo effect. To refute this allegation, we evaluated a series of nutritional and biochemical indices, which are undoubtedly independent of the placebo effect and could provide further information on the effectiveness of the dietary treatment in managing IBS-D patients. Since the variations in the indices mentioned above may only develop slowly over time, the evaluation was performed at baseline and the end of the treatment.

The nutritional variables showed a clear reduction at the end of treatment, demonstrating the effect of participating in a dietary protocol.

This positive effect has already been reported in previous works [8], and its description goes beyond the objectives of this study.

In summary, weight loss and reduced BMI and FM should be considered a consequence of a long-term personalized controlled diet, even if the diet’s energy intake has not been significantly reduced. A significant decrease in FFM occurred at the end of the two diets. This reduction was the consequence of the significant decrease in TBW and ECW. Phase Angle, an indicator of cell membranes’ integrity and water distribution between intra- and extracellular compartments, was unaffected by the diets [33]. Indeed, these variables can be considered unaffected by the placebo effect.

As for the biochemical indicators, all the variables did not change over time, thus indicating that the diets did not affect micronutrients. The ANOVA showed a time effect of some variables, such as blood glucose, CRP, vitamin D, but the post hoc comparisons did not reveal any biological difference. This result confirms that the diet cannot modify the micronutrients even when extended for 12 weeks.

## 5. Conclusions

In agreement with our and others’ previous results, data from the present RCT confirm that LFD significantly improves GI symptoms and QoL in IBS-D patients. However, LFD raises specific issues, such as inadequate nutrient intake without constant dietitian assistance and possible alteration of gut microbiota, worth to be investigated further.

Based on these premises and considering its possible limitations, LFD should be regarded as a first-line dietary approach, necessarily under the control of a dietitian, for those IBS-D patients mainly complaining of bloating, bowel habit dissatisfaction, and interference with QoL.

In light of these results, a valid, equally effective alternative could be considered using a diet based on Tritordeum to manage patients with IBS-D. The results seem promising at present. Notwithstanding, they must be considered preliminary and undoubtedly deserve further confirmation in studies investigating any pathophysiological and intestinal microbiota changes following the adoption of a TBD.

## Figures and Tables

**Table 1:** Energy content and chemical composition of Tritordeum flour and pasta.

Energy Per 100 g	Flour	Pasta
Kcal	350	355.6
Proteins (g)	12.8	12
Fats (g)	1.7	0.1
Saturated fats (g)	0.4	0.0
Dietary fiber (g)	2.6	1.5
Carbohydrates (g)	69.6	75.9
Simple sugars (g)	0.8	4.3
Salt (g)	0.1	0.2

**Table 2:** Ingredients and preparation of Tritordeum-based food.

	Ingredients	Preparation
Pasta	Water and Tritordeum semolina	Long pasta shapes (spaghetti, linguine, and fettuccine) and short pasta shapes (penne rigate and rigatoni) were produced in a pilot pasta-making plant (Intini Food, Putignano-Bari, Italy)
Bread	Tritordeum flour, water, salt, brewer’s yeast	The dough was prepared and risen sufficiently to reach double the volume. Then, it was baked for about 30 min at 240 °C.
Taralli	Tritordeum flour, olive oil, white wine, salt	These salty biscuits were prepared by baking the ready dough for about 40 min at 250 °C.
Breakfast biscuits	Tritordeum flour, lactose-free milk, olive oil, sugar, ammonium bicarbonate	The dough was prepared and then baked for about 20 min at 200 °C.

**Table 3:** IBS Severity Scoring System (IBS-SSS) total score and single-item scores at baseline (V_2_) and after 12 weeks (V_5_) of intervention.

	LFD				TBD				*p*-Value	
Parameters	V_2_	V_3_	V_4_	V_5_	V_2_	V_3_	V_4_	V_5_	Time	Diet × Time
Severity of Abdominal Pain	45.8 ± 23.1	27.9 ± 16.7	29.3 ± 25.1	20.1 ± 21.0	48.7 ± 24.8	27.9 ± 24.2	20.4 ± 19.2	24.4 ± 23.3	<0.0001	0.25
Frequency of Abdominal Pain	45.7 ± 28.0	33.8 ± 28.4	37.1 ± 30.0	19.0 ± 25.5	48.6 ± 31.8	27.8 ± 31.2	26.7 ± 32.8	24.9 ± 26.3	<0.0001	0.68
Severity of Abdominal Distension	51.1 ± 23.9	25.6 ± 17.6	28.2 ± 23.2	23.0 ± 21.3	61.8 ± 21.7	32.1 ± 22.8	29.7 ± 18.8	30.9 ± 24.4	<0.0001	0.10
Dissatisfaction with Bowel Habit	60.2 ± 23.9	41.9 ± 25.7	37.3 ± 25.6	32.3 ± 24.5	69.4 ± 24.9	50.0 ± 26.0	38.6 ± 24.6	41.2 ± 24.3	<0.0001	0.76
Interference with Quality of Life	57.1 ± 21.1	35.8 ± 21.9	33.9 ± 28.8	33.4 ± 27.3	57.5 ± 24.4	40.1 ± 28.7	31.9 ± 23.8	34.0 ± 25.0	<0.0001	0.87

Mixed-design ANOVA analysis and Bonferroni’s multiple comparisons test. LFD = Low-FODMAPs diet; TBD = Tritordeum-based foods. The baseline (V_2_) IBS-SSS scores of each item were significantly (*p* < 0.05) different from the IBS-SSS scores recorded after 4 weeks (V_3_), 8 weeks (V_4_), and 12 weeks (V_5_) of treatment for both diets. Data are expressed as M±SD. Numbers in each group of the IBS-D patients enrolled in the trial were as follows: low-FODMAP diet (LFD) group = 21; Tritordeum based diet (TBD) = 21. The effects on IBS-SSS parameters due to diets were not significant different between the two groups (*p* > 0.05). *p*-Value of “Time” < 0.05 indicated the presence of significant differences between the repeated measures (from V_2_ to V_5_)_,_ irrespective of diet membership. *p*-Value of “Diet × Time” <0.05 indicated the presence of an “interaction effect” with significant differences between the groups and over time. Multiple comparisons: “Severity of abdominal pain” (V_2_ vs. V_5_) *p* < 0.0001 for LFD and TBD. “Frequency of abdominal pain” (V_2_ vs. V_5_) *p* < 0.0001 for LFD, and *p* = 0.0007 for TBD, respectively. “Severity of abdominal distension” (V_2_ vs. V_5_) *p* < 0.0001 for LFD and *p* < 0.0001 for TBD, respectively. “Dissatisfaction with bowel habit” (V_2_ vs. V_5_) *p* = 0.0006 for LFD and *p* = 0.0005 for TBD, respectively. “Interference with quality of life” (V_2_ vs. V_5_) *p* = 0.0012 for LFD and *p* = 0.0015 for TBD, respectively.

**Table 4:** Anthropometric and bioelectrical impedance (BIA) measurements at baseline (V_2_) and after 12 weeks (V_5_) of intervention.

	LFD			TBD			*p* Value	
Parameters	V_2_	V_5_	Change	V_2_	V_5_	Change	Time	Diet × Time
Anthropometric and BIA measurements								
Weight (kg)	64.7 ± 12.9	60.8 ± 12.1	−3.9 ± 2.3	69.4 ± 12.9	66.1 ± 12.0	−3.2 ± 3.1	<0.0001	0.40
BMI (kg/m^2^)	24.3 ± 4.3	23.0 ± 4.2	−1.4 ± 0.9	26.5 ± 4.7	25.2 ± 4.3	−1.3 ± 1.2	<0.001	0.70
Abdominal circumference (cm)	87.0 ± 10.5	84.1 ± 10.9	−2.9 ± 2.5	93.2 ± 10.3	89.9 ± 10.0	−3.2 ± 4.4	<0.001	0.71
Waist Circumference (cm)	77.8 ± 11.7	74.8 ± 10.4	−3.0 ± 2.8	82.8 ± 12.7	79.8 ± 11.7	−2.9 ± 3.0	<0.001	0.90
PhA (degrees)	5.7 ± 0.7	5.9 ± 0.71	0.2 ± 0.4	6.3 ± 1.8	6.5 ± 1.0	0.2 ± 1.2	0.22	0.93
BCM (kg)	24.4 ± 4.8	24.4 ± 5.0	0.0 ± 1.2	26.6 ± 7.7	26.4 ± 6.3	−0.2 ± 2.8	0.73	0.75
FM (kg)	18.0 ± 7.5	15.2 ± 7.1	−2.8 ± 2.0	20.9 ± 7.1	19.4 ± 6.4	−1.5 ± 2.4	<0.001	0.07
FFM (kg)	46.7 ± 8.1	45.7 ± 7.6	−1.0 ± 1.3	48.6 ± 8.4	46.7 ± 8.1	−1.9 ± 2.2	0.0001	0.08
TBW (L)	34.2 ± 6.1	33.3 ± 5.7	0.9 ± 1.1	35.3 ± 5.8	33.8 ± 5.6	−1.5 ± 1.9	0.0002	0.15
ECW (L)	16.1 ± 3.0	15.4 ± 2.4	0.7 ± 1.0	15.9 ± 2.6	14.8 ± 2.1	−1.1 ± 1.6	0.0003	0.43

Mixed-design ANOVA analysis and Bonferroni’s multiple comparisons test. LFD = low FODMAPs diet; TBD = Tritordeum based foods; BMI = Body mass index; PhA = Phase Angle; BCM = Body Cell Mass; FM = Fat Mass; FFM = Free Fat Mass; TBW = Total Body Water; ECW = Extracellular Water. V_2_ = Baseline; V_5_ = 12 weeks of treatment. Data are expressed as M ± SD. Numbers in each group of the IBS-D patients enrolled in the trial were as follows: LFD group = 21; TBD group = 21. The effects on IBS Severity Scoring System parameters due to diets were not significant different between the two groups (*p* > 0.05). *p*-Value of “Time” < 0.05 indicated the presence of significant differences between the repeated measures (V_2_ vs. V_5_)_,_ irrespective of diet membership. *p*-Value of “Diet × Time” < 0.05 indicated the presence of an “interaction effect” with significant differences between the groups and over time. Multiple comparisons: “Weight” (V_2_ vs. V_5_) *p* < 0.0001 for both LFD and TBD. “BMI” (V_2_ vs. V_5_) *p* < 0.0001 for both LFD and TBD. “Abdominal circumference” (V_2_ vs. V_5_) *p* = 0.0088 for LFD and *p* = 0.0030 for TBD, respectively. “Waist Circumference” (V_2_ vs. V_5_) *p* = 0.0001 for LFD and *p* = 0.0002 for TBD, respectively. “FM” (V_2_ vs. V_5_) *p* < 0.0001 for LFD and *p* = 0.0396 for TBD, respectively. “FFM” (V_2_ vs. V_5_) *p* = 0.0711 for LFD and *p* = 0.0001 for TBD, respectively. “TBW” (V_2_ vs. V_5_) *p* = 0.0867 for LFD and *p* = 0.0004 for TBD, respectively. “ECW” (V_2_ vs. V_5_) *p* = 0.1304 for LFD and *p* = 0.0104 for TBD, respectively.

**Table 5:** Biochemical measurements at baseline (V_2_) and after 12 weeks (V_5_) of intervention.

	LFD			TBD			*p* Value	
Parameters	V_2_	V_5_	Change	V_2_	V_5_	Change	Time	Diet × Time
Biochemical profiles								
Total cholesterol (mg/dL)	182.3 ± 34.1	181.6 ± 36.2	−0.8 ± 22.5	195.0 ± 39.60	191.8 ± 33.2	−3.2 ± 30.2	0.63	0.77
HDL cholesterol (mg/dL)	55.9 ± 10.3	55.9 ± 12.6	0.0 ± 7.2	58.0 ± 12.9	55.9 ± 13.2	−3.1 ± 8.1	0.28	0.12
LDL cholesterol (mg/dL)	115.2 ± 41.3	108.4 ± 32.6	−6.8 ± 33.4	117.1 ± 37.3	119.5 ± 31.4	2.4 ± 28.6	0.65	0.35
Triglycerides (mg/dl)	89.3 ± 34.2	86.2 ± 47.4	−3.0 ± 35.1	88.5 ± 42.6	86.8 ± 43.1	−1.7 ± 32.1	0.65	0.90
Total cholesterol/HDL	3.3 ± 0.7	3.3 ± 0.8	0.0 ± 0.6	3.5 ± 1.0	3.7 ± 1.1	0.2 ± 0.5	0.26	0.43
Fasting glucose (mg/dL)	86.0 ± 15.3	79.1 ± 9.2	−6.9 ± 12.5	88.8 ± 12.2	83.8 ± 9.3	−5.1 ± 9.8	0.003	0.62
CRP (mg/dL)	0.2 ± 0.2	0.1 ± 0.1	−0.1 ± 0.2	0.3 ± 0.2	0.2 ± 0.1	−0.1 ± 0	0.02	0.58
Vitamin D (ng/mL)	21.8 ± 8.0	30.8 ± 8.4	9.9 ± 8.8	25.9 ± 17.5	27.4 ± 8.6	1.6 ± 18.5	0.03	0.10
Iron (µg/dL)	104.5 ± 50.3	99.0 ± 36.1	−5.4 ± 50.8	86.0 ± 40.6	113.8 ± 35.4	27 ± 46.3	0.154	0.04
Urea (mg/dL)	32.4 ± 7.1	33.2 ± 5.5	0.8 ± 7.4	30.4 ± 7.5	30.9 ± 6.1	0.5 ± 6.7	0.55	0.90
Creatinine (mg/dL)	0.7 ± 0.2	0.7 ± 0.2	0.0 ± 0.1	0.8 ± 0.2	0.8 ± 0.2	0.0 ± 0.3	0.80	0.24
Urate (mg/dL)	4.3 ± 1.0	4.6 ± 0.8	0.3 ± 1.0	4.9 ± 1.0	4.7 ± 1.0	−0.1 ± 0.7	0.52	0.09
Total protein (g/dL)	7.0 ± 0.4	7.2 ± 0.4	0.1 ± 0.5	7.1 ± 0.4	7.1 ± 0.4	0.0 ± 0.4	0.46	0.24
Cobalamin (pg/mL)	314.1 ± 124.7	327.6 ± 125.9	13.5 ± 67	352.0 ± 187.7	346.4 ± 243.7	−5.7 ± 110.8	0.79	0.51
Folate (ng/mL)	5.9 ± 2.5	6.3 ± 3.3	0.5 ± 1.9	7.5 ± 5.4	6.8 ± 5.7	−0.7 ± 3.9	0.85	0.23

Mixed-design ANOVA analysis and Bonferroni’s multiple comparisons test. LFD = low-FODMAPs diet; TBD = Tritordeum-based foods. V_2_ = Baseline; V_5_ = 12 weeks of treatment. CRP = C-Reactive Protein. Data are expressed as M ± SD. Numbers in each group of the IBS-D patients enrolled in the trial were as follows: LFD group = 21; TBD group = 21. The effects on IBS-SSS parameters due to diets were not significantly different between the two groups (*p* > 0.05). *p*-Value of “Time” < 0.05 indicated the presence of significant differences between the repeated measures (V_2_ vs. V_5_)_,_ irrespective of diet membership. *p*-Value of “Diet × Time” < 0.05 indicated the presence of an “interaction effect” with significant differences between the groups and over time. Multiple comparisons: “Fasting glucose” (V_2_ vs. V_5_) *p* = 0.0675 for LFD, and *p* = 0.3068 for TBD, respectively. “C-reactive protein = CRP” (V_2_ vs. V_5_) *p* = 0.1889 for LFD and *p* = 0.9429 for TBD, respectively. “Vitamin D” (V_2_ vs. V_5_) *p* = 0.0520 for LFD and *p* = 1.0000 for TBD, respectively.

## Data Availability

The datasets used and/or analyzed during the current study are available from the corresponding author on reasonable request.
